# Within- and Across-Species Responses of Plant Traits and Litter Decomposition to Elevation across Contrasting Vegetation Types in Subarctic Tundra

**DOI:** 10.1371/journal.pone.0027056

**Published:** 2011-10-28

**Authors:** Maja K. Sundqvist, Reiner Giesler, David A. Wardle

**Affiliations:** 1 Department of Forest Ecology and Management, Swedish University of Agricultural Sciences, Umeå, Sweden; 2 Department of Ecology and Environmental Science, Climate Impacts Research Centre, Umeå University, Abisko, Sweden; University of Oxford, United Kingdom

## Abstract

Elevational gradients are increasingly recognized as a valuable tool for understanding how community and ecosystem properties respond to climatic factors, but little is known about how plant traits and their effects on ecosystem processes respond to elevation. We studied the response of plant leaf and litter traits, and litter decomposability across a gradient of elevation, and thus temperature, in subarctic tundra in northern Sweden for each of two contrasting vegetation types, heath and meadow, dominated by dwarf shrubs and herbaceous plants respectively. This was done at each of three levels; across species, within individual species, and the plant community using a community weighted average approach. Several leaf and litter traits shifted with increasing elevation in a manner consistent with greater conservation of nutrients at all three levels, and the most consistent response was an increase in tissue N to P ratio. However, litter decomposition was less directly responsive to elevation because the leaf and litter traits which were most responsive to elevation were not necessarily those responsible for driving decomposition. At the community level, the response to elevation of foliar and litter traits, and decomposability, varied greatly among the two vegetation types, highlighting the importance of vegetation type in determining ecological responses to climatic factors such as temperature. Finally our results highlight how understanding the responses of leaf and litter characteristics of functionally distinct vegetation types, and the processes that they drive, to temperature helps provide insights about how future climate change could affect tundra ecosystems.

## Introduction

Elevational gradients can serve as powerful natural experiments for studying how ecological processes are driven by changes in temperature and associated climatic variables, especially when other environmental factors are relatively constant [1], [2]. Further, it is well established that plant functional leaf traits such as leaf and litter nutrient concentrations and leaf mass per unit area can be useful predictors of responses of ecosystem processes to variation in environmental conditions [3], [4], [5]. As temperature is an important factor controlling soil nutrient availability and nutrient supply, some studies have found that foliar nutrient concentrations can decline ([6], [7] but see [8]) and leaf mass per unit area can increase [7], [8] with declining temperatures at higher elevations, at least in tropical and tree line communities [6], [7], [8]. Variation in leaf and litter traits across environmental gradients such as elevation and thus temperature can potentially occur among species, within species, and at the level of the plant community. At the among species level, it is well known that species with trait values such as high leaf dry matter content, low specific leaf area and nutrient content often occur in less productive portions of environmental gradients, for example those involving succession or time since major disturbance [9], [10], [11]. At the within-species level, there is recent and growing recognition that some species which occupy a range of contrasting environmental conditions show significant trait variability across gradients of both soil fertility [11], [12] and macroclimate [4], [13]. The effect of elevation and thus temperature on within-species trait values would be greatest for those species that show a high degree of phenotypic plasticity [14] and that can occupy a large elevational range. With regard to the plant community, some recent studies have shown that community-aggregated trait values (in which each trait in each community is represented by a single value) can be highly responsive to environmental factors [15], [16], [17]. While such an approach has potential for informing how leaf and litter traits may respond to temperature changes such as those associated with increasing elevation at the community level, to our knowledge no study has specifically addressed this.

Foliar leaf and litter traits are important determinants of leaf litter quality [18], [19], [20], and this in turn drives variation in plant litter decomposition rates both among and within biomes [9], [15], [21]. For example, leaf and litter nutrient concentrations are often positively correlated with decomposition rates, while there is often a negative relationship between leaf dry matter content and litter decomposition [21]. Consequently, changes in leaf traits and therefore litter quality with shifts in temperature associated with elevation could potentially have large effects on litter mass loss and nutrient release rates during decomposition in strongly nutrient limited high latitude systems, although this has been seldom explored. However, understanding how the effects of elevation on plant leaf and litter traits across species, within species, and at the community level, in turn influence key ecosystem processes such as decomposition and nutrient fluxes, is important for answering questions about vegetation and ecosystem responses to future climatic change [22]. Further, such changes in litter quality and litter decomposability across gradients of elevation and thus temperature may help explain observed shifts in decomposer communities and soil nutrient fluxes that have been found in several recent studies [23], [24], [25].

Fennoscandian tundra ecosystems contain large gradients of elevation and temperature, and two co-dominant types of vegetation, heath (dominated by woody dwarf-shrubs) and meadow (dominated by herbaceous plants) typically occur at all elevations [26], [27]. These vegetation types are associated with contrasting hydrochemical characteristics, and the heath has lower pH, lower available nitrogen (N), and higher available phosphorus (P) than does the meadow [25], [28], leading to large differences in several above and belowground properties [29]. For example, for the heath vegetation the soil has a higher C:N ratio, and the microbial community has a higher fungal dominance relative to bacteria, compared to the meadow vegetation [25], [29]. Recent work has shown that the response of several belowground properties to increasing elevation and an associated decline in temperature, such as nutrient concentrations and microbial biomass and composition, differs greatly between the two vegetation types through showing unidirectional negative responses for the heath, and idiosyncratic responses for the meadow [25]. However, little is known about how elevation affects leaf and litter traits for contrasting vegetation types, or the consequences for plant litter decomposability. We therefore studied plant leaf and litter traits, and leaf litter decomposability, for both heath (dominating on nutrient poor soils) and meadow vegetation (dominating on more fertile soils), along an elevational gradient in the Swedish subarctic to test three hypotheses.

1. With increasing elevation and thus declining temperature, leaf and litter traits will shift from those associated with rapid nutrient acquisition and high litter quality (and thus high decomposability) to those associated with greater conservation of nutrients and lower litter quality.
2. Shifts in leaf and litter traits, and litter decomposability with elevation will be detectable at each of three levels, i.e., among species, within individual species, and the plant community.
3. Vegetation type (heath versus meadow) will serve as a major driver of how leaf and litter traits, and litter decomposability, respond to elevation and thus changes in temperature.

By addressing these hypotheses we aim to advance our understanding of how leaf and litter characteristics for two functionally distinct vegetation types vary in their response to changes in abiotic factors (notably temperature), and the potential consequences for key decomposer-driven processes that underpin nutrient cycling in subarctic tundra.

## Methods

### Ethics statement

This study was performed across an elevational gradient ranging from 500 to 1000 m, along the North East facing slope of Mt Suorooaivi, located approximately 20 km south-east of Abisko, Sweden (68°21′N,18°49′E), as described by Sundqvist et al. [25]. No areas of the field work occurred within national reserves, and the land is public and not government protected. We confirm that all national and international rules were complied with during the field work. The research did not involve measurements on humans or animals. The plant material collected for this study was only sampled at a very limited scale and therefore had negligible effects on broader ecosystem functioning. We have no commercial interests or conflicts of interest in performing this work.

### Study site and sample collection

In this system there are two co-dominant vegetation types that occur in a mosaic at all elevations, namely heath (dominated by deciduous and evergreen dwarf-shrubs) and meadow (dominated by herbs, graminoids and sedges). In September 2007, four replicate plots (each 10×10 m) were located in each of the vegetation types, at every 100 m along the gradient, yielding a total of 48 plots. The plots were centred on smaller (2 m×2 m) plots used in an earlier study [25]. Plots at the 500 m elevation are situated in open birch forest, plots at 600 m are located immediately above the forest line, and plots from 700 m to 1000 m are devoid of trees. All plots have approximately the same aspect and slope, and parent material is independent of elevation, so in this system climate is the principal abiotic factor that varies with elevation [25]. Measurements using data-loggers on these plots confirm that air temperature during the growing season declines with elevation [25] (Figure S1). Previous work on these plots showed that the heath and meadow vegetation not only differ in several above- and belowground properties, but also in how these properties respond to elevation [25] (Table S1). As such, this system allows us to study how temperature and associated climatic factors that change with elevation affect ecological processes and plant species traits for two contrasting vegetation types that co-occur in the same landscape.

We collected live foliage and fresh litter from between 4 and 6 vascular plant species in each plot. This yielded a total of 18 species; seven species in the heath vegetation and 11 in the meadow vegetation (Table S2). These species were selected to ensure that we included the commonest species at each elevation, as well as those species that occurred across most or all of the gradient, allowing us to effectively explore variation both across and within species across the gradient [11]. For each species we hand-collected at least 10 g of fully expanded green leaves (consisting of at least 30 individual leaves) in each plot between 26 June and 27 July, 2008. For these species we also collected a similar quantity of fresh litter, by hand-collecting senescent leaves still attached to the stem, during September 6–17 2008, and ensured that all of the species collected had reached a comparable stage of peak senescence. All material was air dried (>22°C) after collection, except for leaf samples used for moisture and area measurements as described below.

### Measurements

For each foliar sample, leaf area and fresh weight was determined for 20–35 fresh leaves both immediately before and after oven-drying to constant mass for 48 h. These values were used to calculate leaf dry matter content as the ratio of mass after drying to mass before drying (LDMC; mg g^−1^) and specific leaf area as the ratio of leaf area to remaining mass after drying (SLA; m^2^ kg^−1^). Subsamples of each foliar and fresh litter sample were then ground using a ball mill (Retsch, MM 301) and concentrations of N and P determined by Kjeldahl digestion followed by automated colometric methods (Technicon Instruments 1977). From these measurements ratios of N to P were calculated. For each species collected in each plot we used the concentrations of N and P in fresh litter to estimate nutrient resorption proficiency (percent nutrients in senesced litter) [19], [30].

We assessed the decomposability of each fresh litter for each species collected by using a standardized laboratory bioassay that has been developed specifically for studying the relative decomposability of different litters in a common controlled environment [11], [31]. Here, we used 9 cm Petri dishes each two-thirds filled with a standardized humus substrate (1.5% N, 0.09% P, pH = 3.9), amended to 169% moisture (dry mass basis), collected in mixed spruce and birch forest in Umeå, Sweden (63°50′N, 20°15′E). For each Petri dish a disc of nylon mesh (1 mm holes) was placed on the surface of the soil and fresh litter (1 g, air-dried, and cut into 5 mm fragments when necessary) was placed on the mesh. For each litter sample from each plot we set up three Petri dishes, except when insufficient litter was available in which case only one or two Petri dishes were set up. The Petri dishes were then sealed with tape to minimize water loss and incubated at 22°C for 119 days after which all remaining litter was removed from each Petri dish, rinsed, dried, and weighed. The remaining litter (hereafter referred to as ‘decomposed litter’) in all Petri dishes (normally three) for each litter collection from each plot was then pooled and analyzed for N and P concentration as described above. We determined litter decomposition rate as the percentage mass lost during decomposition. The loss of N and P from the litter was calculated as the total mass × nutrient concentration prior to incubation minus that present after incubation, and expressed as a percentage of the total N or P present at the start of the incubation [31].

### Data analysis

To analyze the effect of elevation on each response variable at the among species level, we calculated the mean value of that variable for each species across all elevations, and the mean elevation for which that species occurred. Linear regression was then used to test for the relationship of each response variable with elevation, with each species serving as an independent data point, as described by Wardle et al. [11]. To study effects of elevation on each response variable at the within species level, we focused on each of five species that occurred on at least five of the six elevations (Table S2), i.e., three from the heath vegetation (*Betula nana*, *Empetrum hermaphroditum* and *Vaccinium vitis-idaea*) and two from the meadow vegetation (*Trollius europaeus* and *Viola biflora*). For each species, data was analysed using one-way ANOVA testing for effects of elevation, and when significant effects were found at *p* (probability) <0.05, differences among means were further analysed by using Tukey's honestly significant difference (h.s.d.) at *p* = 0.05.

To assess the leaf and fresh litter trait and decomposition data at the whole plot level, we calculated plot weighted averages for each variable by weighting all species according to their relative abundance in each plot, which yields a single value for each variable for each plot [15], [16]. This approach requires data on the relative abundance of each species in each plot, and for this study we used abundance data presented by Sundqvist et al. [25] from a 2 m×2 m subplot of each plot used in the present study. This abundance data was obtained by point quadrat analysis [32] through determination of the total number of times the vegetation of that species was intercepted by 100 downwardly projecting points [33]. For each variable, we calculated a weighted average value for each plot by using the following equation [16]:
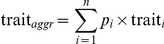



where trait*_aggr_* is the aggregated value of that trait or process for all species collected in that plot, *p_i_* is the cover of species *i* as a proportion of the total cover for all species collected in that plot, and trait*_i_* is the value of the leaf trait or process in species *i*. For each variable, two-way ANOVA was then used to test for the effects of vegetation type and elevation (and their interaction) on the plot weighted average values (i.e., trait*_aggr_*). Whenever ANOVA yielded significant treatment effects of elevation at *p*<0.05, we further analysed differences among means by using Tukey's h.s.d. at *p* = 0.05.

We used correlation analysis to determine the relationship of litter decomposability with all individual leaf and fresh litter traits. These correlations were determined at the plot level, across species level, and within each species. For all data analyses, all data variables were transformed when required to conform to the assumptions of parametric tests. Statistical analyses were performed using the programmed commands for ANOVA, linear regression and correlation analyses provided in the software package SPSS Statistics 17.0.2 (www.spss.com).

## Results

### Effect of elevation among and within species

**Figure 1:**
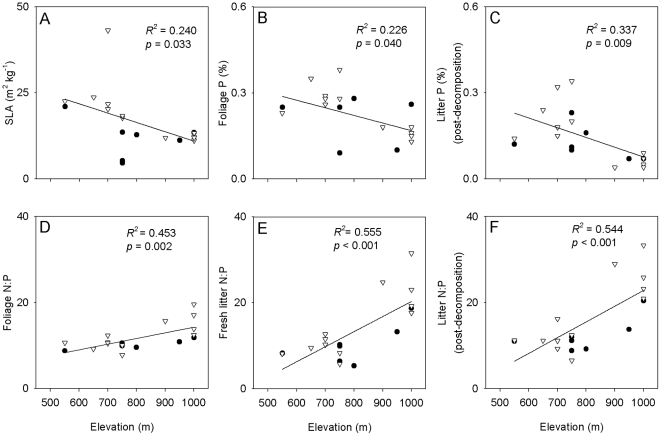
Relationships of leaf and litter traits with elevation across 18 species from heath and meadow. Each species represents a single data point; for each species, the value presented for each leaf and litter trait is the mean for that species across all elevations, and the value presented for elevation is the mean elevation in which that species occurs. Traits are (A) SLA, (B) leaf P concentrations, (C) litter P concentrations post decomposition, (D) leaf N:P ratios, (E) fresh litter N:P ratios and (F) litter N:P ratios post decomposition. White triangles are meadow species; black circles are heath species.

Across species (i.e., each species represented as a single data point), six out of fourteen response variables showed a significant relationship with elevation (Figure 1). As such, SLA had a significant negative relationship with elevation (Figure 1) while LDMC did not (*R* ^2^ (coefficient of determination) = 0.116, *p* = 0.154). Phosphorus showed a significant negative relationship with elevation both for foliage and decomposed litter (Figure 1), but not for fresh litter (*R* ^2^ = 0.194, *p* = 0.059). Across species, elevation showed no significant relationships with N for foliage (*R* ^2^ = 0.003, *p* = 0.823), fresh litter (*R* ^2^ = 0.002, *p* = 0.874) or decomposed litter (*R* ^2^ = 0.108, *p* = 0.170). However, it did show a significant positive relationship with the N to P ratio for all three tissue types (Figure 1). No variable associated with litter decomposability showed a relationship with elevation (*p* always >0.05).

**Figure 2:**
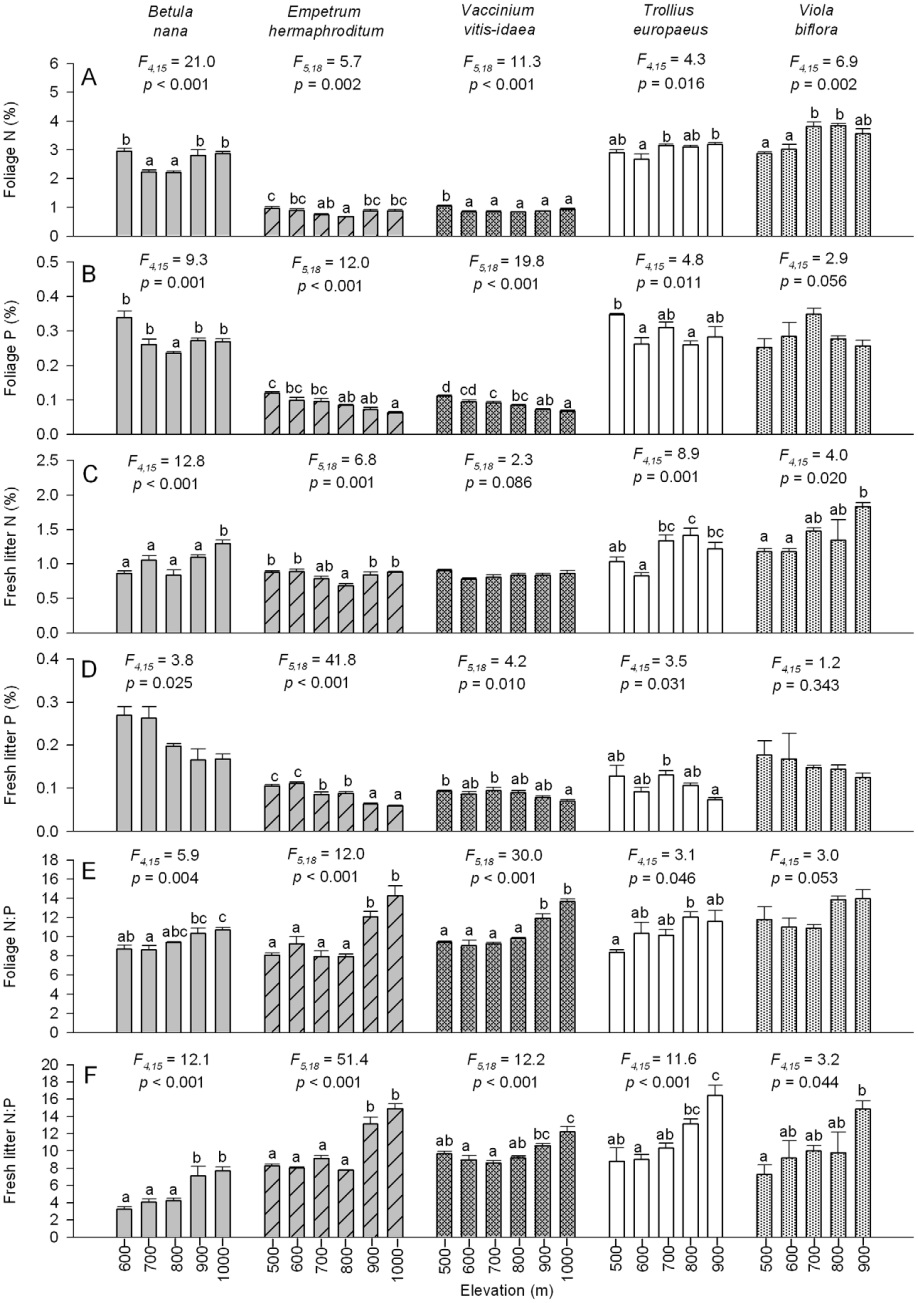
Foliar and fresh litter %N, %P, and their ratios, within species across an elevational gradient. Measures are performed on each of five dominant plant species, three from heath (*B*. *nana*, *E. hermaphroditum* and *V. vitis-idaea*) and two from meadow (*T. europaeus* and *V. biflora*). Measures are (A) foliage N (%), (B) foliage P (%), (C) fresh litter N (%), (D) fresh litter P (%), (E) foliage N:P ratio and (F) fresh litter N:P ratio. For each response variable for each species, *F* and *p*-values (with df) are from a one-way ANOVA testing for the effect of elevation, and bars topped with the same letters are not significantly different at *p* = 0.05 (Tukey's h.s.d.). Note, for fresh litter P (%) for *B. nana* (D) ANOVA yields a significant main effect of elevation but there is no significant difference across elevations according to Tukey's h.s.d.

When analyses were performed within individual species, N and P concentrations were significantly affected by elevation (*p*<0.05) for all three tissue types (foliage, fresh litter, and decomposed litter) of all five species, except for concentrations of N in fresh litter for *V. vitis-idaea* and for concentrations of P for all tissue types for *V. biflora* (Figure 2; data not presented for decomposed litter). Foliage N and fresh litter N were highest at the highest elevations for *T. europaeus* and *V. biflora*, and fresh litter N was highest at the highest elevation for *B. nana*, but foliar and fresh litter N did not show clear unidirectional responses to elevation for the remaining species. Foliage and fresh litter P showed a distinct decline with elevation for *E. hermaphroditum* and *V. vitis-idaea* and to a lesser extent for *B. nana* (and, for fresh litter, *T. europaeus*), but no unidirectional trends emerged for the remaining species. For all five species, the N to P ratios of both foliage and fresh litter were greatest at the highest elevations, and except for *V*. *biflora* foliage this effect was always statistically significant (Figure 2). Generally, litter N and P concentrations and the N to P ratio of decomposed litter showed similar responses to elevation as those of fresh litter (data not presented).

**Figure 3:**
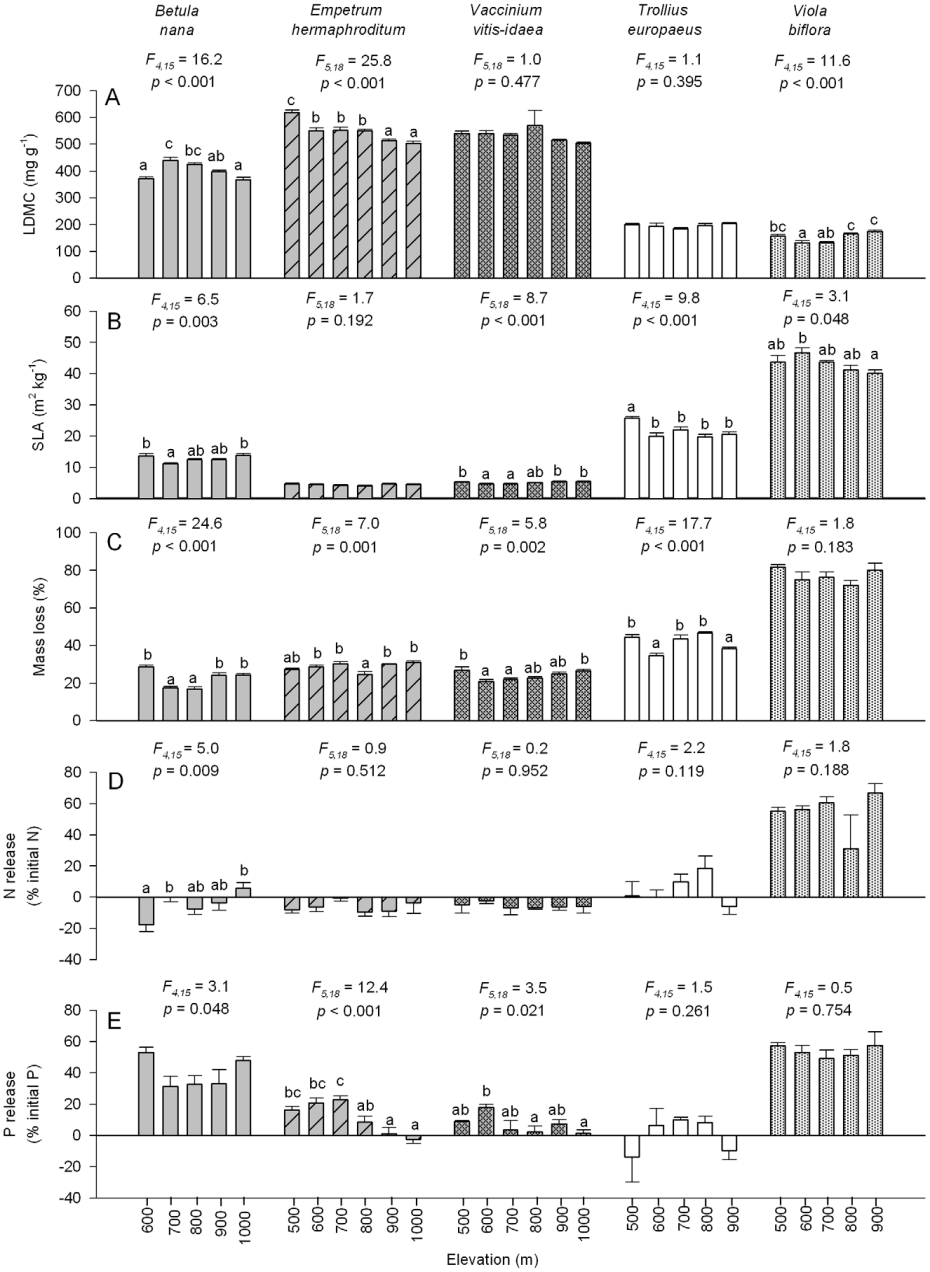
Foliar traits and measures of decomposition within species across an elevational gradient. Measures are performed on each of five dominant plant species, three from heath (*B. nana*, *E. hermaphroditum* and *V*. *vitis*-*idaea*) and two from meadow (*T. europaeus* and *V. biflora*). Measures are (A) leaf dry matter content (LDMC), (B) specific leaf area (SLA), (C) mass loss, (D) N and (E) P loss during decomposition. For each response variable for each species, *F* and *p*-values (with df) are from a one-way ANOVA testing for the effect of elevation, and bars topped with the same letters are not significantly different at *p* = 0.05 (Tukey's h.s.d.). For release of N and P from litter during decomposition, negative values reflect immobilization of N and P. Note, for P release during decomposition (%) for *B. nana* (E) ANOVA yields a significant main effect of elevation but there is no significant difference across elevations according to Tukey's h.s.d.

There were large differences in LDMC and SLA across the five species, and elevation had a significant effect on LDMC for three species and on SLA for four species (Figure 3) but with no consistent pattern with elevation among species. As such, values of LDMC were both highest (for *B. nana*) and lowest (for *V. biflora*) at some intermediate elevations and decreased with elevation for *E. hermaphroditum*. Meanwhile SLA was lowest at some intermediate elevations for *B. nana* and *V. vitis-idaea*, highest at the lowest elevation for *T. europaeus*, and generally declined with elevation for *V. biflora*.

Litter collected at different elevations significantly differed in decomposability, for all species except *V. biflora*, though no species showed a clear unidirectional response to elevation (Figure 3). Release of N from litter during decomposition was only significantly affected by elevation for *B. nana*, largely due to net immobilization of N for litter collected at all elevations except 1000 m (Figure 3). Release of P during decomposition was significantly affected by elevation only for the three heath species, and for two of these (*E. hermaphroditum* and *V. vitis-idaea*) this release was generally lower for litter collected at higher elevations.

### Traits and processes at the whole plot level

**Figure 4:**
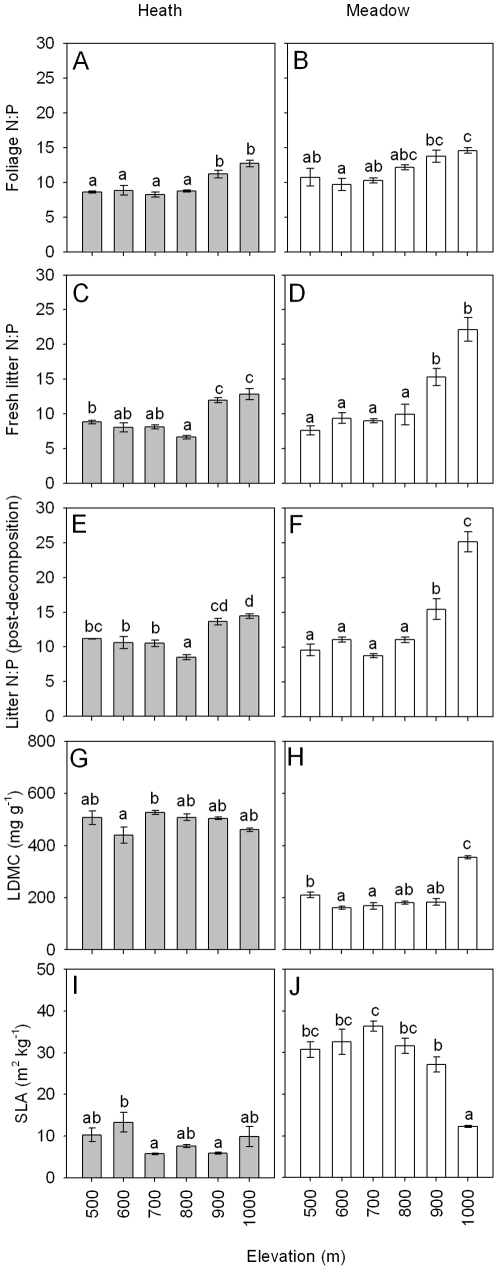
Whole plot foliar and litter traits in heath and meadow vegetation across an elevational gradient. Traits are ratios of N to P in foliage (A–B), fresh litter (C–D), decomposed litter (E–F) and leaf dry matter content (LDMC) (G–H) and specific leaf area (SLA) (I–J) at the whole plot level, with all species collected in each plot weighted according to their relative abundance to provide a single value for each plot. Error bars are standard errors. Within each panel, bars topped with the same letters are not significantly different at *p* = 0.05 (Tukey's h.s.d.). Results from ANOVA testing for differences between the two vegetation types are given in Table 1.

When analyses were done at the whole plot level (i.e., for the community weighted measures, with each plot representing a single data point), concentrations of N and P were significantly higher in meadow than in heath for all tissue types except for P concentrations in fresh litter (Table 1, Table 2). There were also significant effects of elevation on N and P concentrations for all tissue types except for N concentrations in foliage, and significant interactive effects of vegetation type and elevation on N and P concentrations for all tissues (Table 1). For the meadow, intermediate elevations generally had the highest N and P concentrations for all three tissue types, with the lowest concentrations usually at the highest elevation. For the heath, P concentrations did not show a simple unidirectional pattern, but the lowest values occurred at 900 m for foliage, and at 900 and 1000 m for both fresh and decomposed litter, while concentrations of N were generally least at intermediate elevations for all tissue types (Table 2). For all three tissue types, the ratio of N to P was significantly higher for the meadow than for the heath vegetation, and was significantly affected by elevation through always being highest at the two highest elevations (Table 1, Figure 4). For both fresh and decomposed litter, there was a significant interactive effect of vegetation type and elevation on the N to P ratio, because the ratio showed a much larger increase at the highest elevations for the meadow (Figure 4).

**Table 1:** Effect of vegetation type and elevation as revealed by ANOVA (*F* values, with *p* in parentheses) on community-weighted measures of leaf and litter traits and litter mass, N, and P loss during decomposition.

		ANOVA results		
	Variables	Vegetation type (V)	Elevation (E)	V **×** E interaction
Foliage	N (%)^a^	**481.9 (<0.001)**	2.0 (0.107)	**11.6 (<0.001)**
	P (%)^a^	**167.4 (<0.001)**	**11.2 (<0.001)**	**5.8 (<0.001)**
	N:P ratio	**33.4 (<0.001)**	**16.5 (<0.001)**	0.875 (0.508)
	LDMC (mg g^−1^)^a^	**1237.0 (<0.001)**	**20.7 (<0.001)**	**26.7 (<0.001)**
	SLA (m^2^ kg^−1^)^a^	**357.3 (<0.001)**	**8.6 (<0.001)**	**12.4 (<0.001)**
Fresh litter	N (%)^a^	**133.0 (<0.001)**	**3.4 (0.0.012)**	**14.3 (<0.001)**
	P (%)^a^	1.3 (0.262)	**31.2 (<0.001)**	**8.1 (<0.001)**
	N:P ratio^a^	**22.5 (<0.001)**	**34.8 (<0.001)**	**5.0 (0.001)**
Decomposed litter	N (%)	**4444.6 (<0.001)**	**11.3 (<0.001)**	**23.4 (<0.001)**
	P (%)^b^	**81.8 (<0.001)**	**35.5 (<0.001)**	**15.6 (<0.001)**
	N:P ratio^a^	**9.6 (0.004)**	**45.2 (<0.001)**	**11.5 (<0.001)**
Mass loss from decomposition (%)		**584.2 (<0.001)**	**21.3 (<0.001)**	**27.5 (<0.001)**
Release during decomposition:				
	N (% of initial N)^c^	**116.3 (<0.001)**	**3.4 (0.014)**	**4.7 (0.002)**
	P (% of initial P)^c^	**60.0 (<0.001)**	**7.7 (<0.001)**	1.2 (0.310)

Degrees of freedom are 1,36 for V, 5,36 for E and 5,36 for V × E. Values in boldface indicate statistical significance at *p*≤0.05. LDMC is leaf dry matter content; SLA is specific leaf area.

a Data transformed (by *ln* x) before analysis.^ b^Data transformed (by *log* (arcsine x)) before analysis.^c^Data transformed (by √1 + (arcsine x)) before analysis.

**Table 2:** Nitrogen and phosphorus concentrations (%; mean of four plots ± SE) in leaves, fresh litter and post-decomposition litter at the whole plot level in heath and meadow vegetation across an elevational gradient, with all species collected in each plot weighted according to their relative abundance to provide a single value for each plot.

			Elevation (m a.s.l.)					
		Variable	500	600	700	800	900	1000
Heath	Leaf	N (%)	1.4±0.08ab	1.7±0.24b	1.0±0.04a	1.3±0.06ab	1.2±0.04ab	1.5±0.09b
		P (%)	0.16±0.01bc	0.19±0.02c	0.12±0.001ab	0.14±0.01abc	0.11±0.01a	0.13±0.01ab
	Fresh litter	N (%)	0.99±0.02b	0.89±0.04ab	0.84±0.03ab	0.78±0.02a	0.88±0.05ab	0.97±0.004b
		P (%)	0.11±0.004bc	0.12±0.01c	0.12±0.006c	0.14±0.005c	0.08±0.003a	0.09±0.006a
	Litter (post decomposition)	N (%)	1.4±0.05c	1.2±0.02abc	1.2±0.05ab	1.08±0.03a	1.3±0.03bc	1.3±0.04bc
		P (%)	0.12±0.004c	0.12±0.01bc	0.12±0.007c	0.13±0.02c	0.09±0.005ab	0.09±0.007a
Meadow	Leaf	N (%)	2.6±0.04ab	2.9±0.16bc	3.6±0.14d	3.4±0.08cd	3.2±0.20cd	2.3±0.06a
		P (%)	0.26±0.03b	0.32±0.05b	0.35±0.01b	0.29±0.008b	0.24±0.03b	0.16±0.003a
	Fresh litter	N (%)	0.99±0.03a	1.05±0.04a	1.4±0.02b	1.5±0.13b	1.4±0.12b	1.0±0.02a
		P (%)	0.14±0.02bc	0.12±0.009bc	0.16±0.004c	0.16±0.01c	0.10±0.02b	0.05±0.004a
	Litter (post decomposition)	N (%)	2.1±0.1bc	2.1±0.2b	2.5±0.1bc	2.7±0.14c	2.5±0.1bc	1.3±0.04a
		P (%)	0.3±0.06b	0.2±0.02b	0.3±0.005b	0.26±0.01b	0.19±0.03b	0.05±0.005a

ANOVA results shown in Table 1. Within each row, values with the same letters are not significantly different at *p* = 0.05 (Tukey's h.s.d. test).

At the whole plot level, vegetation type, elevation and their interaction also had significant effects on both LDMC and SLA (Table 1). As such, LDMC was higher, and SLA was lower, for heath than for meadow (Figure 4). For the heath, there were no simple unidirectional effects of elevation on either variable. For the meadow, LDMC was highest and SLA was lowest at the highest elevation, and for SLA there was a clear decline with increasing elevation from 700 m (Figure 4).

**Figure 5:**
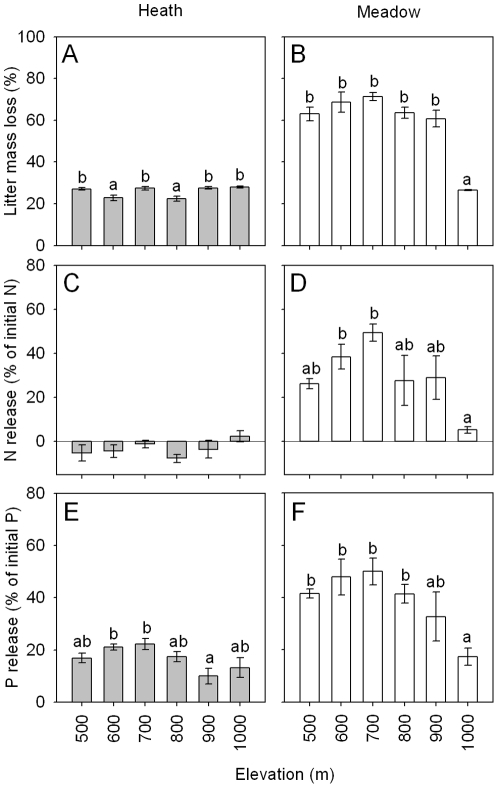
Whole plot measures of decomposition in heath and meadow vegetation across an elevational gradient. Measures are (A–B) litter mass loss, (C–D) N and (E–F) P loss during decomposition calculated at the whole plot level, with all species collected in each plot weighted according to their relative abundance to provide a single value for each plot. Error bars are standard errors. Within each panel, bars topped with the same letters are not significantly different at *p* = 0.05 (Tukey's h.s.d.). For release of N from litter during decomposition, negative values reflect immobilization of N. Results from ANOVA testing for differences between the two vegetation types are given in Table 1.

At the whole plot level, litter decomposability and release of N and P during decomposition, for litters collected in heath and meadow and at different elevations, were both significantly affected by both vegetation type and elevation, and except for P release were also significantly influenced by their interaction (Table 1). The values for each of these variables were, on average, greater for the meadow than the heath (Figure 5). For the heath, litter decomposability and release of N did not show a simple unidirectional relationship with elevation for litter collected at different elevations, while release of P was lowest for litter collected at 900 m and highest for that collected at 600 and 700 m. For the meadow, litter decomposability and N and P release were always lowest for litter collected at the highest elevation (Figure 5).

### Relationships of decomposition with leaf and litter traits

At the across species level (i.e. with each species represented as a single data point), litter decomposability was positively correlated with SLA (*r* = 0.521, *p* = 0.022), but showed no relationship with any other foliar or fresh litter traits (*p* always >0.05). At the within-species level, litter decomposability for *B. nana* were significantly positively related to SLA and leaf N and P concentrations, and negatively related to LDMC (Table 3). Meanwhile for *E. hermaphroditum*, decomposability was significantly positively related to N concentrations in fresh litter and N to P ratios in both foliage and fresh litter. For *V. vitis-idaea*, decomposability was significantly positively related to SLA, and to N concentrations and N to P ratios for both foliage and fresh litter. Litter decomposability for *T. europaeus* was significantly positively related to concentrations of N in fresh litter. No leaf or litter traits were significantly related to litter decomposability for *V. biflora* (Table 3).

**Table 3:** Pearson's correlation coefficients between litter decomposition rate and leaf and fresh litter traits within each of five species across all plots, and at the whole plot level (i.e., with all species collected in each plot weighted according to their relative abundance to provide a single value for each plot).

		Decompostition vs. leaf and fresh litter traits						
		Heath				Meadow		
		*Betula nana* (n = 20)	*Empetrum hermaphroditum* (n = 24)	*Vaccinium vitis-idaea* (n = 24)	Whole plot level (n = 24)	*Trollius europaeus* (n = 20)	*Viola biflora* (n = 20)	Whole plot level (n = 20)
Foliage	N (%)	0.763**	0.352	0.662**	−0.440*	0.339	−0.079	0.504*
	P (%)	0.607**	−0.252	−0.096	−0.556**	0.194	0.081	0.571**
	N:P	0.191	0.527**	0.532*	0.248	0.009	−0.072	−0.338
	LDMC	−0.765**	−0.405	−0.135	0.012	0.123	0.139	−0.401
	SLA	0.512*	0.226	0.522**	−0.316	0.242	−0.016	0.504*
Fresh litter	N(%)	0.178	0.481*	0.595**	0.347	0.612**	−0.017	0.102
	P (%)	0.006	−0.360	−0.187	−0.482*	0.417	0.259	0.328
	N:P	0.120	0.543**	0.531**	0.560**	0.031	−0.100	−0.297

LDMC is leaf dry matter content; SLA is specific leaf area. *, **Correlation coefficient is significantly different to 0 at p = 0.05 and 0.010, respectively. For data at the whole plot level, plots at 1000 m for meadow are excluded from analysis.

At the whole plot level, litter decomposability for the heath was significantly positively related to fresh litter N to P ratios (Table 3), and was also significantly but negatively related to concentrations of leaf N and P, and fresh litter P. Litter decomposability for the meadow plots was significantly positively related to concentrations of leaf N and P, and SLA. No other foliar or litter traits were significantly correlated with litter decomposability for either heath or meadow (*p* always >0.05) (Table 3).

## Discussion

### Shifts in plant functional traits with elevation

Plants that dominate in nutrient poor sites are often assumed to have trait values that differ from those that occur in more favourable sites, for example through having lower SLA, higher LDMC, lower nutrient concentrations and higher nutrient resorption proficiency [10], [34], [35]. We hypothesised that increasing elevation and declining temperature, and associated changes in soil fertility, would cause a shift towards leaf and litter traits associated with greater nutrient conservation at each of three levels, i.e. across species, within species, and at the level of the plant community. We found support for this at the across-species level for only a subset of the traits that we measured; those species that dominated at higher elevations on average had lower SLA and leaf and (decomposed) litter P than those dominating at lower elevations. Further, species dominating at higher elevations generally had greater N to P ratios, indicative of greater limitation of P relative to N [36], [37]. Changing elevation and corresponding shifts in nutrient availability clearly determine the plant species that occur across our study site [25]. Additionally, the results from this study show that those species that dominate at high elevations differ from those at lower levels only with respect to some traits.

Elevation and associated shifts in temperature and soil fertility were also important in influencing leaf and litter traits at the within-species level, and all traits showed significant intraspecific variation for at least some species. We found a consistent (and often strong) positive response of both leaf and litter N to P ratios to increasing elevation for all five species, in much the same manner as found across species, again suggesting greater limitation of P relative to N. However, within species, other trait values only sometimes shifted with increasing elevation in a manner indicative of greater conservation of nutrients and lower litter quality. Further, different species often showed quite different responses to elevation and thus temperature for the same traits (Figure 2 and 3), as well as in the degree of phenotypic plasticity of these traits [14]. Although most plant trait-based literature has focused on across-species variability, our results are consistent with studies highlighting that leaf and litter traits within species that occupy a broad range of environmental conditions can also show considerable variability and large responses to underlying environmental gradients [4], [12], [38].

We further used community-weighted plant functional trait values [16] to evaluate how traits varied across vegetation types and temperature shifts associated with elevation at the whole plot level. We found that plant communities growing on the less fertile heath soils had weighted trait values which were more associated with greater conservation of nutrients when compared to the meadow, such as lower N and P concentration, higher LDMC and lower SLA [15], [39]. In addition, resorption proficiency (percent nutrients in senesced litter), often used as a measure of the ability of plants to withdraw nutrients from leaves before senescence [19], [30], did not differ between the vegetation types for P and was higher in the meadow than heath for N. These findings generally support previous data from this study system [25] that the nature of nutrient limitation differs between the two vegetation types, with limitation of N relative to P being greater for the heath than for the meadow.

In earlier work on this study system the response of several belowground properties to increasing elevation and thus declining temperature was found to differ greatly for heath and meadow vegetation [25] (Table S1). We therefore hypothesized that vegetation type would serve as a major driver of responses of community-weighted (whole plot) values of leaf and litter traits to elevation and associated shifts in temperature and soil fertility. However, while our results often pointed to differences in the responses of the two vegetation types to elevation, there were relatively few instances in which effects of elevation on trait values were unidirectional, and in most cases these occurred for the meadow. For example the increase in litter N to P ratios with elevation was much more pronounced for the meadow than for the heath, and SLA showed a strong decline for the meadow only. As such, our findings support that community-weighted values of plant functional traits can be strongly responsive to environmental gradients [15], [17], [20]. However, they also reveal that the same traits may respond very differently to the same elevational gradient when different vegetation types are involved, and with herbaceous vegetation associated with soil fertility showing stronger unidirectional responses.

### The effect of elevation on decomposition processes

Leaf and litter traits are well known to be linked to the quality of litter for decomposer organisms, and can therefore be important predictors of decomposition and nutrient release [18], [20], [21]. We therefore predicted that shifts in leaf and litter trait values towards those indicative of greater nutrient conservation with higher elevation and thus declining temperature, would exert negative effects on litter quality and thus decomposability. At the across species level, there was no relationship between decomposition (or N or P release) and elevation, despite there being a significant correlation between decomposition and SLA, and SLA in turn being related to elevation. At this level, decomposition was not correlated with any other trait that varied with elevation, and our prediction was unsupported. Within species, several leaf and fresh litter traits were correlated with mass loss, but different traits were important in driving mass loss for different species (Table 3), pointing to high variability among species in trait-decomposition relationships [40]. Litter decomposability had significant relationships with elevation for all but *V. biflora*, but these relationships were never unidirectional. Tissue N to P ratios, which were the traits most consistently responsive to elevation and thus temperature, showed significant relationships with mass loss for only *E. hermaphroditum* and *V. vitis-idaea*, and otherwise decomposition was mostly related to traits that did not show clear unidirectional relationships with elevation (Figure 3; Table 3). However, we found that the release of P from decomposing litter declined with elevation for *E. hermaphroditum* and *V. vitis-idaea*, suggesting that nutrient release from decomposing litter can show a simple relationship with elevation even when mass loss does not. While our results show that within-species trait variability can be important as a driver of decomposition [11], [41] they also highlight that decomposition will respond in a simple way to extrinsic environmental factors only when the traits that drive it also show a simple response to those factors [42].

At the community-level (using community-weighted values), the heath vegetation (dominating on poorer soils [25] and characterized by low SLA and N concentrations, and high LDMC) produced litter that lost mass, N and P more slowly than litter from the meadow, which is consistent with expectations that plant communities on less fertile soils produce poorer quality litter [9], [15]. However, we found large differences between the two vegetation types in terms of which traits were important in driving decomposition at the community-level. Across the meadow plots, mass loss was positively correlated with leaf traits commonly associated with higher litter quality, and litter from the highest elevations also decomposed and released nutrients the slowest. In contrast, litter mass loss across the heath plots was mostly related to traits that were not strongly related to elevation, and therefore there was no simple relationship between decomposition and elevation. While our results show that shifts in functional traits at the community-level can serve as determinants of litter decomposition across environmental gradients [15], [17], they also highlight that relationships between plant traits, litter decomposability and environmental gradients can differ greatly among contrasting vegetation types which differ in soil fertility. In particular, our results provide evidence that, at the community-level, litter quality and decomposability are more responsive to environmental gradients across which there is a greater shift in plant community composition and thus in the turnover of species (see [11]), which is the case here for the meadow relative to the heath [25].

### Conclusions

In our study we sought to test three hypotheses. For the first one, we found that an increase in elevation and associated shifts in temperature and soil fertility often caused shifts in leaf and litter traits in a direction associated with greater conservation of nutrients and lower litter quality, but these shifts were not consistently related to a decrease in litter mass loss and nutrient release. For the second, we revealed that shifts in leaf and litter traits with elevation and thus temperature could occur at each of three levels, i.e., across species, within species, and at the plant community-level, with the most consistent trend across all three levels involving increasing foliar and/or litter N to P ratios with increased elevation. For the third, we found that leaf and litter traits, and loss of litter mass, N and P during decomposition were often more responsive to elevation for the meadow than for the heath vegetation. These results in combination have several implications. First, they highlight that shifts in leaf and litter traits of tundra plants in response to decreasing elevation and increasing temperature do not necessarily result in increasing mass and nutrient loss rates during decomposition. This may be because those traits that are influenced the most by temperature are not necessarily those that drive decomposition, or because the magnitude of change in trait values in response to temperature is not sufficient to impact on decomposability. Second, vegetation type (in this case, heath versus meadow) can be a powerful determinant of how extrinsic environmental factors such as temperature affect plant functional traits, litter decomposability and ultimately ecosystem processes. Third, they provide evidence that, in tundra ecosystems, increasing elevation is a major driver of tissue N to P ratios across species, within species and at the whole plot level, meaning that decreasing temperature causes a consistent shift in the availability of P relative to N. Finally, our results emphasize that understanding of the responses of dominant tundra species, and functionally contrasting vegetation types, to changes in abiotic factors such as temperature and soil fertility is essential for predicting how future climate change might affect ecosystem processes in subarctic tundra.

## Supporting Information

Figure S1 **Daily mean air temperature (°C), June 28 - August 31 2008, at the study site.** From [25] with permission from John Wiley and Sons.
(TIF)

Table S1 **Plant community characteristics and soil abiotic factors (mean ± se) for heath and meadow across an elevational gradient.**
(DOC)

Table S2 **List of species sampled for this study, their functional group and at which elevations and in which vegetation type they were sampled.**
(DOC)
