# Supplementary material for: Within- and Across-Species Responses of Plant Traits and Litter Decomposition to Elevation across Contrasting Vegetation Types in Subarctic Tundra
Source: PLoS One. 2011 Oct 28;6(10):e27056. doi: 10.1371/journal.pone.0027056 (PMC3203947; doi:10.1371/journal.pone.0027056)
Supplement: Table S1 — Plant community characteristics and soil abiotic factors (mean ± se) for heath and meadow across an elevational gradient. (DOC) [file pone.0027056.s002.doc]

**Table S1**. Plant community characteristics and soil abiotic factors (mean ± se) for heath and meadow across an elevational gradient.

|  |  | Vegetation characteristics | | Soil characteristics | | | | | |
| --- | --- | --- | --- | --- | --- | --- | --- | --- | --- |
| Vegetation | Elevation | Species richness | Vegetation density | Soil moisture (%) | Ammonium (mg g^-1^ dw) | Phosphate (mg g^-1^ dw) | C:N | C:P | N:P |
| Heath | 500 | 8.0 ± 1.68abc | 178.0 ± 17.55 | 66.2 ± 4.6a | 0.024 ± 0.002a | 0.038 ± 0.005a | 31.9 ± 1.4a | 493.7 ± 92.1 | 15.7 ± 3.1ab |
|  | 600 | 7.25 ± 0.48abc | 172.25 ± 23.48 | 69.8 ± 1.9a | 0.021 ± 0.002a | 0.01 ± 0.001c | 23.7 ± 1.4b | 395.4 ± 24.0 | 16.8 ± 1.3ab |
|  | 700 | 6.5 ± 0.96bc | 169.25 ± 24.43 | 69.4 ± 1.9a | 0.009 ± 0.001b | 0.025 ± 0.004ab | 29.2 ± 0.5a | 410.1 ± 17.6 | 14.0 ± 0.4b |
|  | 800 | 7.75 ± 0.25 c | 168.75 ± 6.54 | 64.8 ± 4.8a | 0.011 ± 0.004b | 0.017 ± 0.002b | 30.6 ± 0.8a | 450.8 ± 20.3 | 14.7 ± 0.3 ab |
|  | 900 | 9.75 ± 0.75 a | 178.75 ± 10.70 | 38.5 ± 6.3b | 0.004 ± 0.001c | 0.007 ± 0.002cd | 22.6 ± 1.4b | 391.0 ± 34.2 | 17.2 ± 0.4ab |
|  | 1000 | 8.5 ± 0.65ab | 160.5 ± 6.89 | 48.2 ± 9.5ab | 0.005 ± 0.001c | 0.005 ± 0.001d | 24.2 ± 1.3b | 448.4 ± 38.5 | 18.5 ± 1.2a |
| Meadow | 500 | 22.5 ± 2.06ab | 237.5 ± 11.72a | 76.3 ± 1.7 | 0.027 ± 0.003ab | 0.012 ± 0.003a | 13.8 ± 0.8a | 292.7 ± 49.1a | 15.7 ± 1.9ab |
|  | 600 | 24.25 ± 2.78a | 237.25 ± 26.52a | 66.1 ± 4.0 | 0.064 ± 0.04ab | 0.006 ± 0.001ab | 15.7 ± 0.4b | 216.4 ± 14.6bc | 13.8 ± 1.1bc |
|  | 700 | 19.0 ± 0.82bc | 145.5 ± 16.21b | 70.8 ± 1.4 | 0.043 ± 0.003ab | 0.009 ± 0.0004a | 14.9 ± 0.4b | 173.5 ± 9.0c | 11.6 ± 0.35 |
|  | 800 | 25.75 ± 0.95a | 140.5 ± 9.19b | 60.0 ± 1.4 | 0.009 ± 0.003c | 0.006 ± 0.0009ab | 15.5 ± 0.8b | 215.1 ± 8.3bc | 14.0 ± 1.0bc |
|  | 900 | 22.75 ± 1.44ab | 178.75 ± 12.57b | 62.0 ± 12.9 | 0.054 ± 0.015a | 0.014 ± 0.004a | 16.2 ± 0.3b | 251.5 ± 16.0ab | 15.6 ± 1.0ab |
|  | 1000 | 17.0 ± 0.71c | 130.5 ± 16.65b | 54.62 ± 6.8 | 0.019 ± 0.002bc | 0.004 ± 0.0006b | 16.1 ± 0.4b | 279.3 ± 18.4a | 17.3 ± 0.72 |

Data derived from Sundqvist et al. [25]. Within each row, values with the same letters are not significantly different at *p* = 0.05 (LSD test with Benjamini-Hochberg correction). Vegetation density is determined by point quadrat analysis and represents the total number of intercepts for 100 downwardly projecting points [32].

**Table S2**. List of species sampled for this study, their functional group and at which elevations and in which vegetation type they were sampled.

| Species | Functional group | Elevation (m a.s.l.) | Vegetation type |
| --- | --- | --- | --- |
| *Bartsia alpina* | Herbaceous | 600 – 900 | Meadow |
| *Betula nana* | Deciduous dwarf-shrub | 600-1000 | Heath |
| *Bistorta vivipara* | Herbaceous | 1000 | Meadow |
| *Carex aquatilis* ssp. *stans* | Sedge | 900 | Meadow |
| *Carex bigelowii* | Sedge | 1000 | Heath and Meadow |
| *Carex saxatilis* | Sedge | 1000 | Meadow |
| *Cassiope tetragona* | Evergreen dwarf-shrub | 900 – 1000 | Heath |
| *Empetrum hermaphroditum* | Evergreen dwarf-shrub | 500 – 1000 | Heath |
| *Geranium sylvaticum* | Herbaceous | 500 – 600 | Meadow |
| *Saussurea alpina* | Herbaceous | 500 – 600, 800 – 900 | Meadow |
| *Salix polaris* | Deciduous dwarf-shrub | 1000 | Meadow |
| *Sibbaldia procumbens* | Herbaceous | 700 – 800 | Meadow |
| *Solidago virgaurea* | Herbaceous | 500 – 800 | Meadow |
| *Trollius europaeus* | Herbaceous | 500 – 900 | Meadow |
| *Vaccinium myrtillus* | Deciduous dwarf-shrub | 500 – 600 | Heath |
| *Vaccinium uliginosum* | Deciduous dwarf-shrub | 600 – 900 | Heath |
| *Vaccinium vitis-idaea* | Evergreen dwarf-shrub | 500 – 1000 | Heath |
| *Viola biflora* | Herbaceous | 500 – 900 | Meadow |
