# Supplementary material for: Within- and Across-Species Responses of Plant Traits and Litter Decomposition to Elevation across Contrasting Vegetation Types in Subarctic Tundra
Source: PLoS One. 2011 Oct 28;6(10):e27056. doi: 10.1371/journal.pone.0027056 (PMC3203947; doi:10.1371/journal.pone.0027056)
Supplement: Table S2 — List of species sampled for this study, their functional group and at which elevations and in which vegetation type they were sampled. (DOC) [file pone.0027056.s003.doc]

**Table S2**. List of species sampled for this study, their functional group and at which elevations and in which vegetation type they were sampled.

| Species | Functional group | Elevation (m a.s.l.) | Vegetation type |
| --- | --- | --- | --- |
| *Bartsia alpina* | Herbaceous | 600 – 900 | Meadow |
| *Betula nana* | Deciduous dwarf-shrub | 600-1000 | Heath |
| *Bistorta vivipara* | Herbaceous | 1000 | Meadow |
| *Carex aquatilis* ssp. *stans* | Sedge | 900 | Meadow |
| *Carex bigelowii* | Sedge | 1000 | Heath and Meadow |
| *Carex saxatilis* | Sedge | 1000 | Meadow |
| *Cassiope tetragona* | Evergreen dwarf-shrub | 900 – 1000 | Heath |
| *Empetrum hermaphroditum* | Evergreen dwarf-shrub | 500 – 1000 | Heath |
| *Geranium sylvaticum* | Herbaceous | 500 – 600 | Meadow |
| *Saussurea alpina* | Herbaceous | 500 – 600, 800 – 900 | Meadow |
| *Salix polaris* | Deciduous dwarf-shrub | 1000 | Meadow |
| *Sibbaldia procumbens* | Herbaceous | 700 – 800 | Meadow |
| *Solidago virgaurea* | Herbaceous | 500 – 800 | Meadow |
| *Trollius europaeus* | Herbaceous | 500 – 900 | Meadow |
| *Vaccinium myrtillus* | Deciduous dwarf-shrub | 500 – 600 | Heath |
| *Vaccinium uliginosum* | Deciduous dwarf-shrub | 600 – 900 | Heath |
| *Vaccinium vitis-idaea* | Evergreen dwarf-shrub | 500 – 1000 | Heath |
| *Viola biflora* | Herbaceous | 500 – 900 | Meadow |
